# Particle-Assisted Dermal Penetration—A Simple Formulation Strategy to Foster the Dermal Penetration Efficacy

**DOI:** 10.3390/pharmaceutics14051039

**Published:** 2022-05-11

**Authors:** Sabrina Wiemann, Cornelia M. Keck

**Affiliations:** Department of Pharmaceutics and Biopharmaceutics, Philipps-University of Marburg, Robert-Koch-Str. 4, 35037 Marburg, Germany; sabrina.wiemann@pharmazie.uni-marburg.de

**Keywords:** fluorescein, dermal penetration, ex vivo porcine ear model, digital image analysis, stratum corneum thickness, skin hydration

## Abstract

(1) Background: The study systematically investigated the influence of dispersed particles within a topical formulation on the dermal penetration efficacy of active compounds that are dissolved in the water phase of this formulation. The aim was to prove or disprove if particle-assisted dermal penetration can be used for improved dermal drug delivery. (2) Methods: Fluorescein was used as a surrogate for a hydrophilic active ingredient (AI). It was dissolved in the water phase of different formulations with and without particles. Two different types of particles (titanium dioxide and nanostructured lipid carriers (NLC)) were used. The influence of particle size and number of particles and the influence of skin hydrating excipients was also investigated. (3) Results demonstrate that the addition of particles can strongly increase the dermal penetration efficacy of AI. The effect depends on the size of the particles and the number of particles in the formulation, where smaller sizes and higher numbers resulted in higher penetration parameters. Formulations with NLC that contained 20% *w*/*w* or 40% *w*/*w* particles resulted in an about 2-fold higher amount of penetrated AI and increased the penetration depth about 2.5-fold. The penetration-enhancing effect was highly significant (*p* < 0.001) and allowed for an efficient delivery of the AI in the viable dermis. In contrast, the penetration-enhancing effect of excipients that increase the skin hydration was found to be very limited and not significant (≤5%, *p* > 0.05). (4) Conclusions: Based on the results, it can be concluded that particle-assisted dermal penetration can be considered to be a simple but highly efficient and industrially feasible formulation principle for improved and tailor-made dermal drug delivery of active compounds.

## 1. Introduction

**Figure 1:**
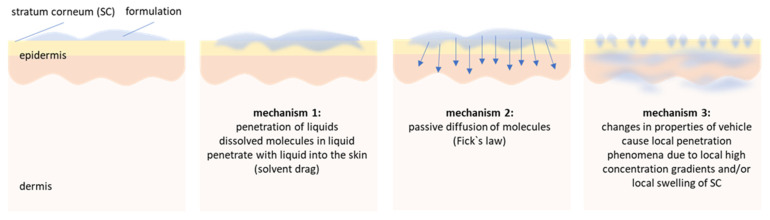
Proposed mechanisms of dermal penetration. First mechanism: penetration of low viscous liquids—active compounds penetrate via convection (solvent drag mechanisms) into the skin. Second mechanism: vehicle and skin are in equilibrium—AI penetrate via passive diffusion. Third mechanism: transformation of vehicle creates passive penetration phenomena—one example is the particle-assisted penetration enhancement, i.e., the formation of aqueous menisci below particulate materials that increase the penetration of AI being dissolved in the liquid of the meniscus [3]. It is likely that the different mechanisms occur not stepwise but superimpose each other. The figure and the figure legend are re-printed from [2].

In addition to oral drug delivery, today, dermal drug delivery is one of the most often exploited administration routes. In dermal drug delivery, the dermal penetration of active ingredients (AI) is considered to take place by passive diffusion that can be explained by Fick’s law. Hence, after topical application of a formulation, the AI is considered to diffuse to the interface between the vehicle and the skin from where it enters the skin [1]. However, recent findings now demonstrated that the dermal penetration of chemical compounds cannot only be explained by Fick’s law and showed that at least two additional mechanisms must be considered when predicting the dermal penetration efficacy of AI after topical application [2,3]. The first additional penetration mechanism is the penetration of AI via convection, and the second additional mechanism is particle-assisted penetration (Figure 1). 

The penetration of AI via convection, i.e., via a solvent drag mechanism, means that low viscous liquids from the vehicle can penetrate into the skin. Consequently, AI that are dissolved in these liquids will enter the skin together with their solvent [2]. The second new mechanism is particle-assisted penetration (mechanism 3, Figure 1). The mechanism was recently shown for drug nanocrystals, i.e., nanosuspensions that are composed of poorly water soluble AI that are dispersed as particles in submicron size in aqueous media [4,5,6,7,8,9].

**Figure 2:**
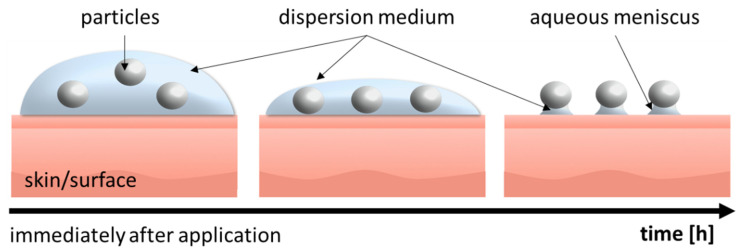
Scheme of aqueous meniscus formation after topical application of particle-containing dermal formulations.

Particle-assisted dermal penetration is considered to occur if a topical formulation contains particles that are dispersed in a liquid that can evaporate and/or penetrate the skin after topical application. Hence, after topical application, the volume of dispersion medium decreases over time and finally connects the particles to the surface of the skin via an aqueous meniscus (Figure 2). The aqueous meniscus causes a prolonged attachment of the particles on top of the skin. The increased retention time of the particles enhances the dermal penetration for the active compounds that are released from these particles. This is not only due to the longer retention time, but also due to the formation of a locally high concentration gradient of dissolved AI within the aqueous meniscus, and in addition, due to a local swelling, i.e., increased hydration of the stratum corneum (SC), which can further foster the dermal penetration of the AI [3].

Until now, the improved dermal penetration of AI due to the formation of an aqueous meniscus was only shown for drug carriers, i.e., for dermal formulations that contained particles with incorporated AI. However, based on the previous findings we hypothesized that particle-assisted dermal penetration, i.e., the formation of an aqueous meniscus, should also allow for an improved dermal penetration of AI that are not incorporated into the particles but that are only dissolved in the dispersion medium. The aim of this study was therefore to prove or disprove this theory and to investigate to which extent dermal penetration of dissolved AI can be enhanced by particle-assisted dermal penetration, i.e., by the addition of particles to topical formulations.

The study was performed with two different types of particle dispersions (titanium dioxide (TiO_2_) and nanostructured lipid carriers (NLC)) that were dispersed in different aqueous dispersion media. Sodium fluorescein is a hydrophilic fluorescent dye. It was dissolved in the water phase of the particle dispersions and served as a surrogate for a hydrophilic AI. The different formulations were prepared and characterized, and the dermal penetration efficacy of the AI surrogate from the different particle-containing formulations was determined with the ex vivo porcine ear model [10,11]. The results obtained were compared to the dermal penetration of the AI surrogate from formulations with identical dispersion media without particles. 

## 2. Materials and Methods

### 2.1. Materials

Fluorescein disodium salt as hydrophilic AI surrogate was obtained from JT Baker Inc., Phillipsburg, NJ, USA. TiO_2_ particles were obtained from Titan Kogyo Ltd., Ube, Japan. NLC were produced in house (cf. Section 2.2.3). The lipid matrix of the NLC consisted of 60% *w*/*w* cetyl palmitate and 40% *w*/*w* medium-chain triglycerides (Miglyol^®^ 812). Both lipids were obtained from Caesar & Loretz GmbH, Hilden, Germany. Alkylpolyglucosides (Plantacare^®^ 818 UP and Plantacare^®^ 2000 UP, BASF AG, Ludwigshafen, Germany) were used as stabilizers for the particles. The more hydrophilic Plantacare 818 was used for the stabilization of the NLC and the more hydrophobic Plantacare 2000 was used for the stabilization of the more hydrophobic TiO_2_ particles, respectively. Glycerol (≥97%) was used as humectant and was obtained from VWR International GmbH, Leuven, Belgium. Purified water was freshly obtained daily from a PURELAB^®^ Flex 2 (ELGA LabWater, Veolia Water Technologies GmbH, Celle, Germany). 

### 2.2. Methods

#### 2.2.1. Study Design

The study was performed in three steps. In the first step, particle-assisted dermal penetration was investigated with titanium dioxide (TiO_2_) particles. For this, TiO_2_ particles (2% *w*/*w*) were dispersed in water or surfactant solution (Plantacare 2000 3% *w*/*w*). The AI surrogate (0.005% *w*/*w*) was dissolved in these formulations and particle-free formulations with similar dispersion media served as controls (Table 1). The distribution of the particles within the formulations was investigated with light microscopy. In addition, the Feret diameter of the dispersed particles was determined. The formulations were applied on fresh porcine skin and the dermal penetration efficacy was determined. 

In the second step, particle-assisted dermal penetration was investigated with NLC. The NLC were produced by high-pressure homogenization (HPH) and their physical–chemical properties, i.e., size and zeta potential, were analysed. NLC were produced with different lipid contents (5%, 10%, 20%, 40%, Table 1) and 0.005% *w*/*w* AI surrogate was dissolved in the water phase of these formulations. An NLC-free formulation (particle-free formulation) that consisted of 1% *w*/*w* surfactant (Plantacare^®^ 818 UP) and 0.005% *w*/*w* AI surrogate served as benchmark control (Table 1). All formulations were applied on fresh porcine skin and their dermal penetration efficacy was determined.

The last part of the study investigated if the addition of humectants that are known to increase the skin hydration can be used to further improve the particle-assisted dermal penetration efficacy. For this, 5% *w*/*w* glycerol was added to the NLC formulation with 10% lipid content and 0.005% *w*/*w* AI surrogate. The resulting physical–chemical properties, i.e., size and zeta potential of the formulation with and without glycerol, were analysed and the dermal penetration efficacy of the AI surrogate was determined ex vivo on fresh porcine skin. Formulations without particles and glycerol served as controls (Table 1). A detailed description of the production of the formulations, their characterization, and dermal penetration efficacy testing is provided below. 

#### 2.2.2. Production and Characterization of TiO_2_ Dispersions

Purified water and a 3% Plantacare 2000 solution served as dispersion media for TiO_2_. The latter was prepared by adding the emulsifier to purified water by constant stirring at room temperature for 10 min. The AI surrogate fluorescein was added to both media and stirred until it was completely dissolved. For the suspensions, TiO_2_ was placed in a beaker, the respective aqueous phase was added, and the mixture was stirred (150 rpm) for 5 min. The particle dispersions were analysed by light microscopy equipped with a polarization filter (Olympus BX53, Olympus Corporation, Shinjuku, Japan; magnification: 200-fold). Subsequently, the images obtained were subjected to digital image analysis (Image J software, version 1.53 a, National Institute of Health, Gaithersburg, MD, USA) [12,13] to estimate the particle size, i.e., the Feret diameter, of the dispersed particles. For this, the scale of the software was set to 3.3 µm/px. The images were converted into 8-bit images and subjected to a black/white threshold that allowed to discriminate and select particles from the background of the image. The selected threshold included all pixels with a brightness ≥80 grey values. Finally, the marked particles were analysed by the ImageJ software [14]. Only particles ≥0.4 µm were included in the analysis, as smaller areas were considered to be not concrete particles but artefacts, because such small particles cannot be detected by ordinary light microscopy. The Feret diameters for all particles obtained were transferred into JASP software (version 14.1, Universiteit van Amsterdam, Amsterdam, The Netherlands [15]) to calculate the mean Feret diameters [16]. 

#### 2.2.3. Production of the NLC Dispersions

The NLC are a well-known drug delivery system with superior properties on skin [17,18,19,20,21,22,23,24]. In this study, they were produced after a previously established production protocol [25]. The lipids were molten at 75 °C while stirring constantly. The aqueous phase was heated likewise and added to the lipid phase while stirring. The obtained pre-dispersion was subjected to high-speed stirring (Ultra Turrax T25, IKA-Werke GmbH & Co. KG, Staufen im Breisgau, Germany) for 30 s at 10,000 rpm and subsequently to hot high-pressure homogenization by using a LAB 40 (APV Gaulin GmbH, Lübeck, Germany) in discontinuous mode for 3 cycles at 500 bar and at a temperature set to 75 °C. The so obtained hot nanoemulsions were filled into Falcon tubes that were immediately placed in an ice bath to cool the dispersions to yield the NLC. The NLC were characterized and used for dermal penetration testing within 24 h of production.

#### 2.2.4. Characterization of the NLC Dispersions

The particle size of the NLC was determined by three independent techniques [26]. Dynamic light scattering (DLS, Zetasizer Nano ZS, Malvern Panalytical Ltd., Malvern, UK, data analysis in general purpose mode) was used to determine the mean particle size, i.e., the hydrodynamic diameter, of the NLC dispersions. Laser diffractometry (LD, Mastersizer 3000, Malvern Panalytical Ltd., Malvern, UK, data analysis with Mie theory with optical parameters set to 1.5 (real refractive index) and 0.001 (imaginary refractive index)) was used to prove or disprove the absence of possible larger particles that cannot be determined by DLS measurements [26]. In addition, light microscopy (Olympus BX53, Olympus Corporation, Shinjuku, Japan; magnification: 400-fold and 1000-fold) was used to confirm the data obtained by LD measurements [26]. In addition to size, the zeta potential (ZP, Zetasizer Nano ZS, Malvern Panalytical Ltd., Malvern, UK) was analysed. The analysis was performed at 20 °C in the original dispersion medium (surfactant solution) and in purified water with adjusted conductivity with sodium chloride (50 µS/cm, 20 °C). The zeta potential was determined by measuring the electrophoretic mobility (EM) by Laser Doppler Anemometry. The EM obtained was then converted into the ZP by using the Helmholtz–Smoluchowski equation [27].

#### 2.2.5. Determination of Dermal Penetration Efficacy

The influence of the particles on the dermal penetration efficacy was investigated by using the ex vivo pig ear model [10,11]. Fresh pig ears were obtained from a local slaughterhouse from pigs that were slaughtered for food industry. Ears were washed with lukewarm water and gently dried with a paper towel. The hairs were carefully trimmed to 1–3 mm with scissors without touching the skin. To ensure skin integrity, the transepidermal water loss was measured (TEWL, Tewameter^®^ TM300, Courage + Khazaka Electronic GmbH, Köln, Germany). A TEWL value of >15 g/cm^2^/h was defined as an exclusion criterion. Subsequently, examination areas of 2 × 2 cm, which showed no visible injuries, were marked on the ears. An amount of 50 µL of the respective formulation (cf. Table 1) were applied and evenly distributed over the area using the saturated finger method [28]. The ears were incubated for 1 h in a pre-heated oven at 32 °C. After the penetration time, the excess formulation was carefully removed with rinsing water, and the ears were carefully blotted dry in dabbing movements with a lint free tissue. Punch biopsies (Ø 15 mm) were taken from each examination area, embedded in Tissue Tec^®^ (Sakura Finetek Europe BV, Alphen aan den Rijn, The Netherlands), and frozen at −80 °C. For further analysis, the punch biopsies were cut into 20 µm thick vertical skin sections using a cryomicrotome (Mod. 2700 Reichert-Jung, Nußloch, Germany). 

The skin cuts were placed on microscopic slides and the dermal penetration of the AI surrogate was evaluated by inverted epifluorescence microscopy (Olympus CKX53, equipped with an Olympus DP22 colour camera, Olympus Life Science Solutions GmbH, Hamburg, Germany). The filter block system DAPI HC was used for analysis (excitation filter: 460–490 nm (BP), dichroic mirror 500 nm, emission filter: starting at 500 nm (LP)). The exposure time was set to 50 ms, and the intensity of the fluorescent light source (130 W U-HGLGPS illumination system, Olympus Deutschland GmbH, Hamburg, Germany) to 50%. The magnification of the microscope was 200-fold. All settings were kept constant throughout the study.

#### 2.2.6. Digital Image Analysis

Digital image analysis was used to assess the penetration parameters, i.e., the relative amount of penetrated AI surrogate and the corresponding penetration depth from the microscopic images [11]. For this, the images from fluorescence microscopy were subjected to an automated RGB threshold algorithm (Supplementary Material Section S1) that subtracted the autofluorescence of the skin from the fluorescence of the AI surrogate by using ImageJ software (version 1.53 a, National Institute of Health, USA) [12,13]. The remaining autofluorescence of the images after the RGB threshold (ART) is expressed as mean grey value per pixel (MGV/px). The ART corresponds to the amount of penetrated AI surrogate and is therefore used to evaluate the amount of penetrated active compounds into the skin semi-quantitatively [11]. The penetration depth of the AI surrogate was determined from the thresholded images with the scale function of ImageJ software. The scale was set to 2.84 px/µm and the penetration depth was measured for each image. The mean penetration depth (MPD) is the average of all measured images and is expressed as mean ± standard deviation (SD). Like the MPD, the stratum corneum thickness (SCT) was assessed from the original images. From the SCT, the relative SCT (rel. SCT in %) was calculated by setting the SCT of untreated skin (control) to 100%. 

#### 2.2.7. Statistical Analysis

Statistical analysis was performed with JASP software (version 14.1., Universiteit van Amsterdam, Amsterdam, The Netherlands [15]). Normal distribution of the data was checked with the Shapiro–Wilk test and variance homogeneity was determined with the Levene’s test. One-way analysis of variance (ANOVA) with Welch adoption in case of variance inhomogeneity was conducted for normally distributed data. A Kruskal–Wallis H test was performed for non-parametric data sets. Appropriate post hoc tests (Tukey, Games–Howell or Dunn’s post hoc test [29]) and independent t-tests were also performed to determine significant differences between the different mean values. All *p*-values < 0.05 were considered statistically significant.

## 3. Results

### 3.1. Particle-Assisted Dermal Penetration with Titanium Dioxide Particles

**Figure 3:**
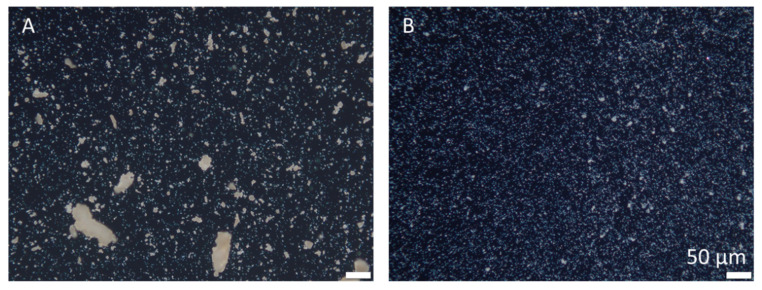
Microscopic images of the TiO_2_/water dispersion (**A**) and the TiO_2_/surfactant dispersion (**B**). Images were taken with the polarization filter and with a 200-fold magnification.

Dispersion of the TiO_2_ in water resulted in the formation of an inhomogeneous suspension with large TiO_2_ aggregates (Figure 3A). The dispersion of the TiO_2_ particles in the surfactant solution resulted in a more homogenous distribution of the particles with smaller agglomerates (Figure 3B). The Feret diameter of the TiO_2_/water dispersion was 3.84 µm and was 2.98 µm for the TiO_2_/surfactant dispersion, and both dispersions possessed very broad size distributions. The differences in size were significant (independent *t*-test, *p* < 0.001). 

**Figure 4:**
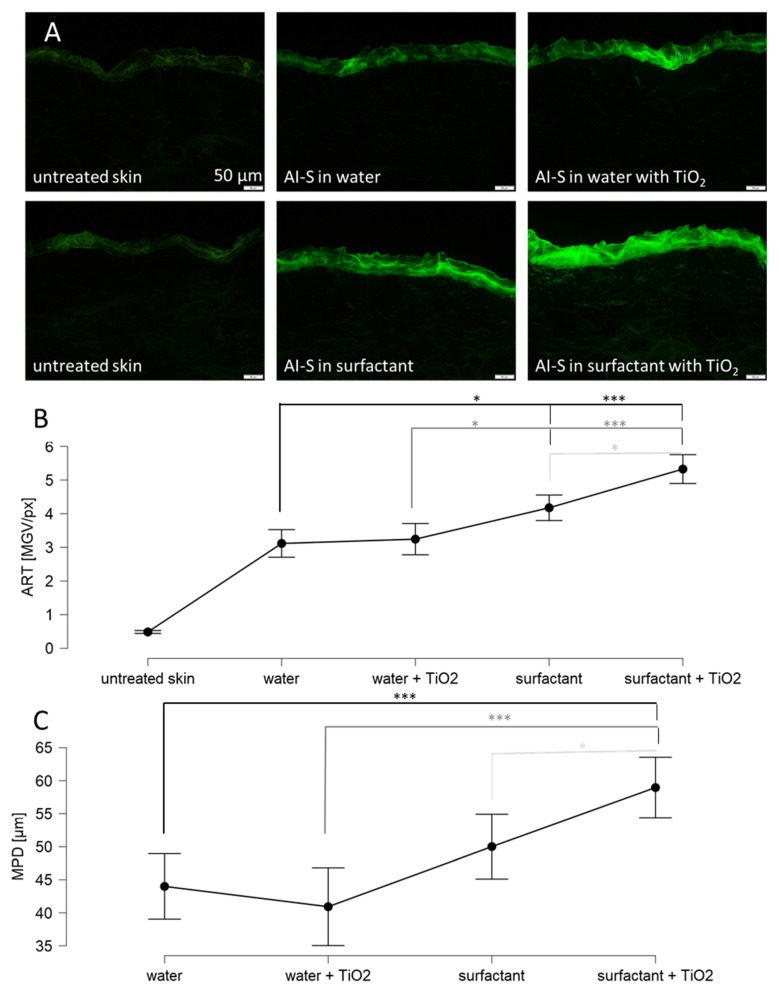
Dermal penetration efficacy of skin treated with sodium fluorescein solution (in water or surfactant) with and without TiO_2_ particles. (**A**) images of vertical skin sections obtained by inverted epifluorescence microscopy, (**B**) ART values as surrogate for the total amount of penetrated AI, (**C**) mean penetration depth (MPD). AI-S = AI surrogate = fluorescein. Untreated was significant in relation to all measured values. The asterisks indicate the following *p*-values: * ≤0.05; *** ≤0.001.

The dermal penetration efficacy of the AI surrogate was affected by the type of dispersion medium and by the addition of the particles (Figure 4). The addition of surfactant increased the penetration of the AI surrogate significantly by about 35% and the penetration depth was increased by about 15%. The addition of TiO_2_ particles to the water did not affect the penetration efficacy of the AI surrogate but was significantly increased when the TiO_2_ particles were dispersed in the surfactant solution (Figure 4). The increase was about 30% for the total amount of penetrated AI and about 20% for the penetration depth (Figure 4B,C).

The penetration-enhancing effect of the surfactants on the dermal penetration efficacy was expected, because surfactants are considered to modify the structure of the SC, which can cause an enhanced permeability and penetration efficacy of AI [30,31,32,33,34,35]. Particle-assisted penetration was not observed for the large-sized TiO_2_ particles in water but was nicely demonstrated for the smaller sized and more homogenously distributed TiO_2_ particles in the surfactant solution. The controversial results for the particle-assisted dermal penetration are also reasonable. The aqueous meniscus connects the particles to the skin. The curvature of the meniscus that is created between skin and particle and the resulting adhesive forces between particle and skin depend on the size of the particles [36]. Based on the Kelvin equation, with decreasing particle size, the radius of curvature decreases and the adhesive forces increase. Hence, the large size of the TiO_2_ particles in water can be considered to hamper particle-assisted penetration, whereas the size of the TiO_2_ particles in the surfactant solution seemed to be sufficiently small to allow for effective particle-assisted dermal penetration. In addition to the size, the total number of particles within the dispersion also might impact the dermal penetration efficacy, i.e., less particles can be considered to create less menisci and thus will result in less effective particle-assisted dermal penetration. The large agglomerates of TiO_2_ in water resulted in a much smaller number of particles in the dispersion and thus caused a much smaller number of particles that were connected to the skin. Hence, both parameters, the low number and the larger size of the particles in water, can be considered to be responsible for the non-observed particle-assisted penetration when TiO_2_ particles were dispersed in water.

### 3.2. Particle-Assisted Dermal Penetration with NLC

**Figure 5:**
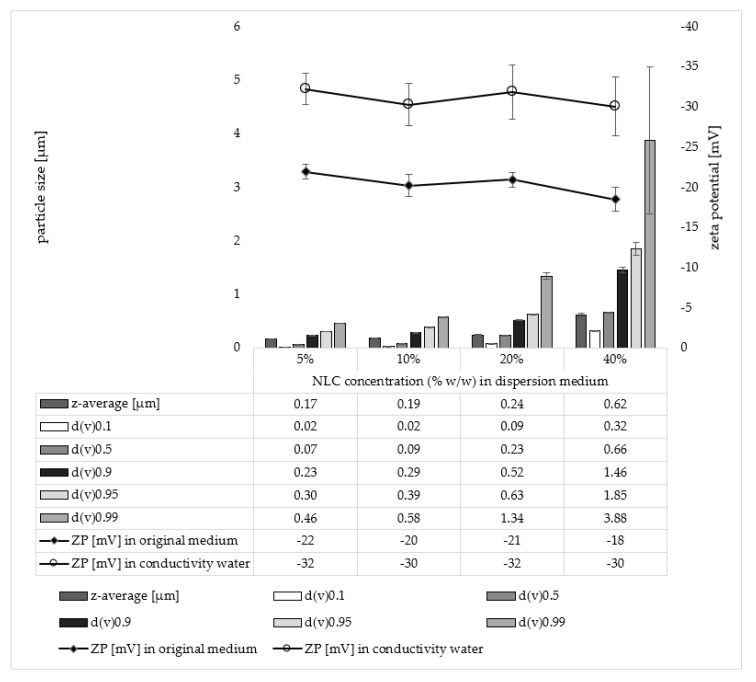
Influence of lipid content of NLC on particle size and zeta potential (ZP) of the NLC formulations. The particle size was assessed with dynamic light scattering which yields the hydrodynamic diameter (z-average). Laser diffraction was used to determine the volume-based size distribution of the particle dispersions. The obtained median volumetric diameters d(v)0.1–d(v)0.99 (µm) represent the percentage of the volume of the particles within the dispersion that are equal or below the given size. Zeta potentials (mV) were analysed in the original medium and in conductivity water, respectively.

**Figure 6:**
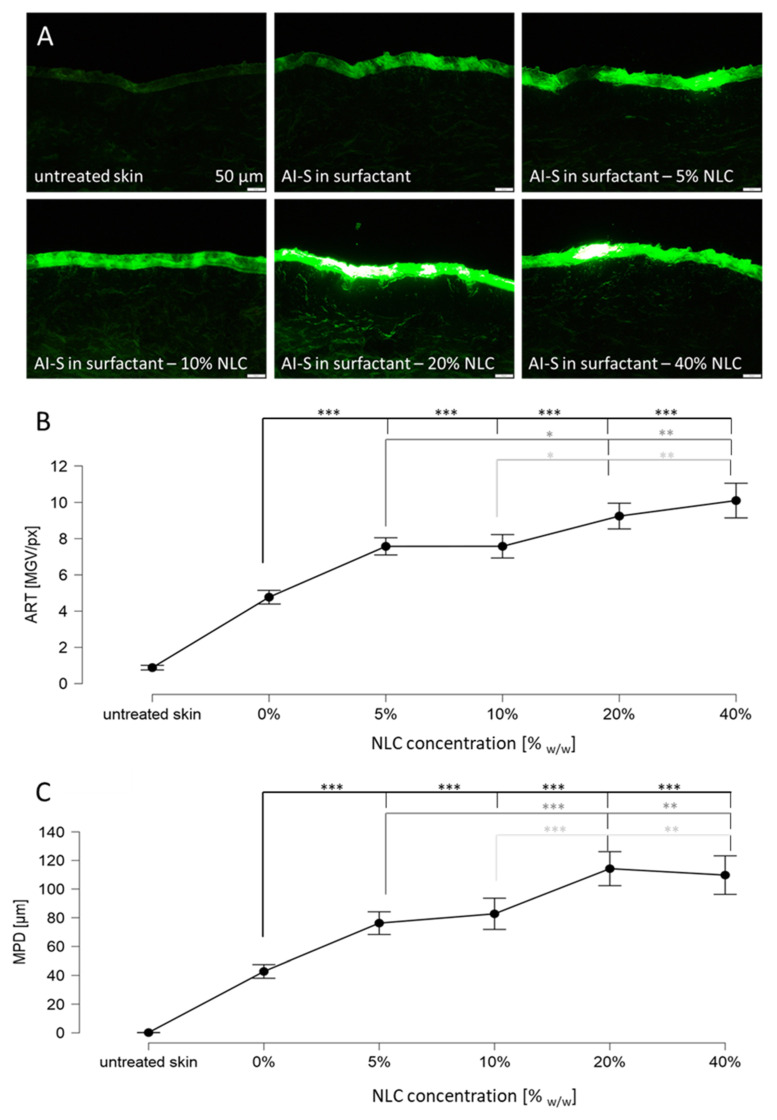
Dermal penetration efficacy of skin treated with sodium fluorescein solution with and without NLC particles. (**A**) Images of vertical skin sections obtained by inverted epifluorescence microscopy, (**B**) ART values as surrogate for the total amount of penetrated AI, (**C**) mean penetration depth (MPD). AI-S = AI surrogate = fluorescein. NLC = nanostructured lipid carriers. Untreated was significant in relation to all measured values. The asterisks indicate the following *p*-values: * ≤0.05; ** ≤0.01; *** ≤0.001.

The data obtained from the TiO_2_ particles already showed that the dermal penetration efficacy of hydrophilic AI can be enhanced by the addition of particles. Data indicated that the size and number of the particles impact the particle-assisted dermal penetration. Therefore, the next part of the study aimed at investigating these effects in more detail. For this, NLC with different lipid concentrations, i.e., particle contents, were produced, and their dermal penetration efficacy was investigated and compared to a formulation without particles. An increase in lipid concentration from 5 to 40% resulted in a significant increase in particle size and size distribution but had no significant impact on the ZP (Figure 5). Particle-assisted dermal penetration was observed for all NLC formulations (Figure 6). 

The addition of 5% NLC increased the amount of penetrated AI surrogate by about 60% and the penetration depth was increased by about 80%. Addition of 10% particles increased the amount of penetrated AI surrogate by about 60% and increased the penetration depth by about 90%. The addition of 20% NLC increased the amount of penetrated AI surrogate by about 95% and resulted in an increase in the penetration depth to about 265% when compared to the formulation without particles. Addition of 40% NLC resulted in an increase in the amount of penetrated AI surrogate by about 110% and increased the penetration depth to about 255%. The results between 5% and 10% and between 20% and 40% are not significantly different. This means that the results can be clustered into three groups. Group I is the formulation without particles. Group II clusters the formulations with low particle content in the range between 5–10%, and group III clusters the formulations with high particle content, i.e., formulations with particle concentrations in the range between 20–40%. Significant differences in both, the amount of penetrated AI surrogate and the penetration depth, were found between these groups. The results clearly demonstrate that an increasing number of particles results in an increased dermal penetration of active compounds. They also show that the effect is not only limited to the total amount of penetrated AI but is also affecting its penetration depth. 

The application of fluorescein solution without particles led to a mean penetration depth of about 43 µm. This corresponds approximately to the thickness of the stratum corneum. Hence, the application of fluorescein solution without particles (group I) only enabled a penetration of fluorescein into the SC. The addition of low particle concentrations (group II) resulted in a mean penetration depth of about 80 µm, and the addition of high particle concentrations (group III) resulted in a mean penetration depth of about 110 µm. The average thickness of the epidermis is about 100 µm. Hence, low particle concentrations allowed the fluorescein to penetrate into the epidermis and the addition of high particle concentrations even allowed for a transdermal penetration. Data therefore show that the addition of particles not only affects the total amount of penetrated AI but also the region in the skin to which the active compound can be delivered. Data therefore suggest that the addition of particles cannot only be used for enhanced dermal drug delivery but also to produce formulations with tailor-made penetration profiles. 

### 3.3. Particle-Assisted Penetration and Addition of Humectants

The first parts of the study clearly showed that particles in dermal formulations can strongly affect the dermal penetration of active compounds that are dissolved in the water phase of the formulation. In addition to particle-assisted dermal penetration, which was demonstrated in this study, other parameters can also affect the dermal penetration efficacy. One important parameter that is known to improve the dermal penetration efficacy is the skin hydration [37]. As effective dermal penetration of the AI is desired for most of the topical formulations, it was interesting to investigate if an increase in skin hydration due to the addition of humectants can further increase the dermal penetration of particle-containing formulations. Therefore, in the last part of the study, glycerol (which is a natural moisturizing factor and well-known humectant) was added to the dispersion medium with and without particles and the dermal penetration efficacy was determined accordingly. 

**Figure 7:**
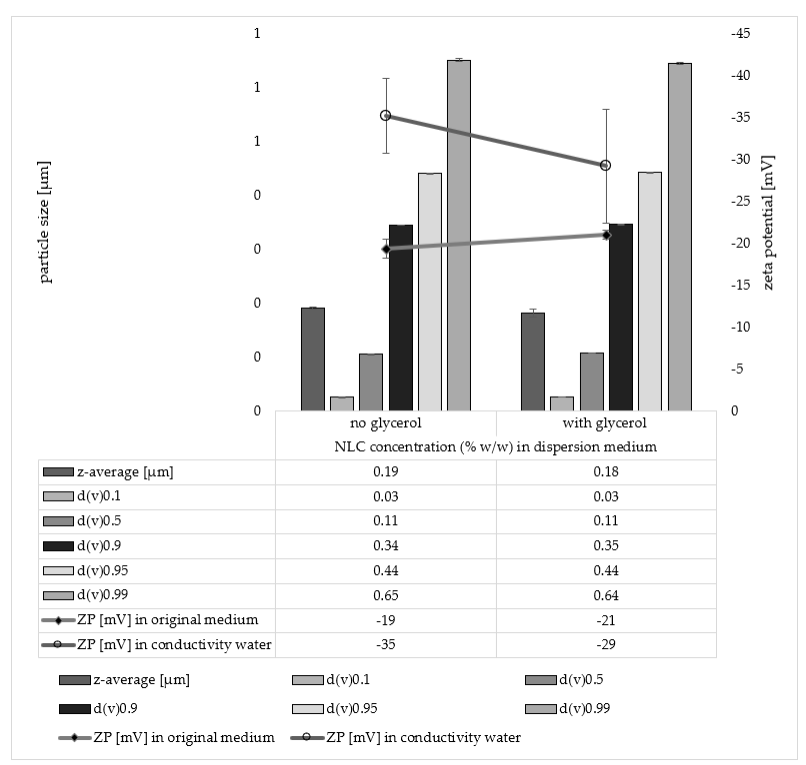
Influence of glycerol on particle size and zeta potential of the NLC dispersion. The particle size was assessed with dynamic light scattering which yields the hydrodynamic diameter (z-average). Laser diffraction was used to determine the volume-based size distribution of the particle dispersions. The obtained median volumetric diameters d(v)0.1–d(v)0.99 (µm) represent the percentage of the volume of the particles within the dispersion that are equal or below the given size. Zeta potentials (mV) were analysed in the original medium and in conductivity water, respectively.

The addition of glycerol to the particles had no influence on the particle size. The ZP seemed to be slightly affected but the changes were not significant (Figure 7). 

**Figure 8:**
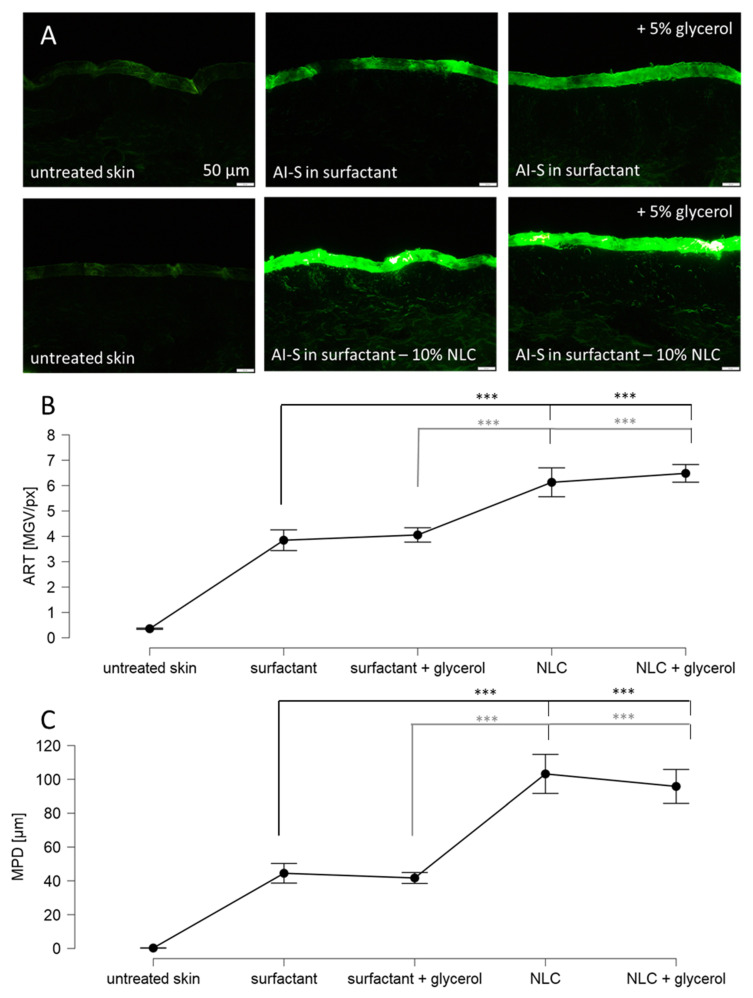
Dermal penetration efficacy of skin treated with sodium fluorescein surfactant solution with and without NLC particles and with and without glycerol. (**A**) Images of vertical skin sections obtained by inverted epifluorescence microscopy, (**B**) ART values as surrogate for the total amount of penetrated AI, (**C**) mean penetration depth (MPD). AI-S = AI surrogate = fluorescein. NLC = nanostructured lipid carriers. Untreated was significant in relation to all measured values. The asterisks indicate the following *p*-value: *** ≤0.001.

The addition of glycerol to the surfactant solution without particles increased the amount of penetrated AI surrogate by about 5% (Mann–Whitney test, *p* > 0.05) and had no influence on the penetration depth (Figure 8). The addition of particles increased the amount of penetrated AI surrogate by about 60% and the penetration depth by about 130%. The addition of glycerol to the particle-containing formulation increased the penetrated amount of the AI surrogate by about 5% (Mann–Whitney test, *p* > 0.05) and had no influence on the penetration depth. 

Therefore, data indicate the addition of humectants and particles is additive but not synergistic. Data also demonstrate that the particle-assisted penetration has a much greater impact on the all-over dermal penetration of active compounds than the addition of moisturizers (Figure 8). 

**Figure 9:**
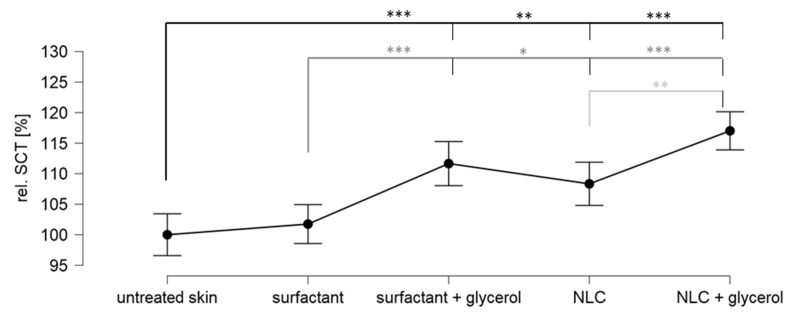
Relative stratum corneum thickness of skin treated with sodium fluorescein in surfactant solution with and without NLC particles and with and without glycerol. The asterisks indicate the following *p*-values: * ≤0.05; ** ≤0.01; *** ≤0.001.

The weak influence of glycerol on the dermal penetration efficacy was not expected and it was therefore speculated that the expected skin hydrating effect of glycerol was maybe not achieved in this experiment. Therefore, to investigate this assumption and to provide a more detailed understanding on the influence of glycerol and the particles on the dermal penetration efficacy, the stratum corneum thickness of the differently treated skin sections was analysed (Figure 9). 

The surfactant solution had no influence on the SCT. The addition of glycerol increased the SCT by about 10% and the addition of particles increased the SCT by about 5%. The addition of particles and glycerol increased the SCT by about 15%. Data show that the addition of glycerol caused a strong increase in skin hydration that was evidenced by the increase in SCT. The addition of particles also caused an increase in skin hydration. However, the effect was only half as strong as the addition of glycerol. The addition of glycerol and particles caused an additive hydration effect. 

A possible explanation why the strong hydration of glycerol was not effective to improve the dermal penetration of the AI surrogate might be related to the hygroscopic properties of glycerol. If glycerol is only able to penetrate into the SC but not into deeper layers of the skin, it might hold water in the SC but at the same time it might soak water from deeper layers of the skin or at least prevent the penetration of water from the formulation into deeper layers of the skin. Consequently, the hydrophilic AI surrogate that is dissolved in the water also cannot penetrate deeper into the skin. More research is needed to understand these mechanisms in more detail. 

## 4. Conclusions

The results prove that particles in a topical formulation can tremendously increase the dermal penetration efficacy of active compounds that are dissolved in the water phase of this formulation. The so-called particle-assisted penetration depends on the particle size and the number of particles within the formulation. Higher numbers and smaller sizes increase the total amount of penetrated active compound. The increase in penetration efficacy seems to reach a plateau once the skin surface is fully covered with particles. In this case, higher numbers of particles cannot further improve the penetration efficacy of the active compound. In this study, NLC with particle concentrations between 20% *w*/*w*–40% *w*/*w* in the formulation were identified to be most effective. They resulted in an about 2-fold higher penetration when compared to the formulation without particles. 

Particles in topical formulations were also found to strongly impact, i.e., increase, the penetration depth. This means active compounds that only penetrate into upper skin layers if formulated in classical, particle-free formulations can be efficiently transported into deeper layers of the viable dermis with the help of particles that are added to these classical formulations. The use of different numbers of particles and or particle sizes might even allow for the formulation of topical products with tailor-made penetration depth in the future. More research is needed to address these findings in more detail. 

The addition of humectants (glycerol) resulted in a significant increase in skin hydration but had only a limited effect on the dermal penetration efficacy of the AI surrogate. The penetration-enhancing effect of glycerol was additive to the particle-assisted dermal penetration enhancement but not synergistic. Finally, the data obtained allowed for a direct comparison between the penetration-enhancing effects that are caused by skin hydration (addition of humectant) and the addition of particles. The limited effect of the humectant (approx. +5%, no effects on the penetration depth) and the pronounced increase in dermal penetration efficacy (approx. +100%, approx. 2.5-fold deeper penetration) upon the addition of particles provide sound evidence that particle-assisted dermal penetration can be considered to be a novel, simple, and super-effective formulation principle for improved and tailor-made dermal drug delivery of active compounds. The principle is industrially feasible. It requires no additional equipment and is easily scalable. On the other hand, it should also be considered that the addition of particles might also foster the penetration of other compounds, i.e., preservatives, perfumes, etc., that can promote allergic and/or toxic reactions. More research is now needed to exploit and investigate the principle and potential of particle-assisted penetration to real world formulations. 

## Figures and Tables

**Table 1:** Overview of formulations and compositions of the formulations tested.

**1. Particle-assisted dermal penetration with titanium dioxide particles**			
***Formulation code***	***particles***	***dispersion medium***	***AI surrogate***
1-water-control	-	water	0.005% *w*/*w*
1-TiO_2_ in water	TiO_2_—2% *w*/*w*	water	0.005% *w*/*w*
1-surfactant control	-	surfactant 3% *w*/*w*	0.005% *w*/*w*
1-TiO_2_ in surfactant	TiO_2_—2% *w*/*w*	surfactant 3% *w*/*w*	0.005% *w*/*w*
**2. Particle-assisted dermal penetration with NLC**			
***Formulation code***	***particles***	***dispersion medium***	***AI surrogate***
2-NLC-control	-	surfactant 1% *w*/*w*	0.005% *w*/*w*
2-NLC 5%	NLC *—5% *w*/*w*	surfactant 1% *w*/*w*	0.005% *w*/*w*
2-NLC 10%	NLC *—10% *w*/*w*	surfactant 1% *w*/*w*	0.005% *w*/*w*
2-NLC 20%	NLC *—20% *w*/*w*	surfactant 1% *w*/*w*	0.005% *w*/*w*
2-NLC 40%	NLC *—40% *w*/*w*	surfactant 1% *w*/*w*	0.005% *w*/*w*
**3. Particle-assisted penetration and addition of humectants**			
***Formulation code***	***particles***	***dispersion medium***	***AI surrogate***
3-surfactant control	-	surfactant 1% *w*/*w*	0.005% *w*/*w*
3-glycerol control	-	surfactant 1% *w*/*w*, glycerol 5% *w*/*w*	0.005% *w*/*w*
3-NLC control	NLC *—10% *w*/*w*	surfactant 1% *w*/*w*	0.005% *w*/*w*
3-NLC with glycerol	NLC *—10% *w*/*w*	surfactant 1% *w*/*w*, glycerol 5% *w*/*w*	0.005% *w*/*w*

* The lipid matrix of the NLC consisted of a mixture of cetyl palmitate and medium-chain triglycerides in a ratio 6:4.

## Data Availability

Data are contained within the article and Supplementary Material Section S1.

## Supplementary Materials

The following supporting information can be downloaded at: https://www.mdpi.com/article/10.3390/pharmaceutics14051039/s1, Section S1: Macros used for the automated RGB threshold for the digital image analysis.
