# Supplementary material for: Particle-Assisted Dermal Penetration—A Simple Formulation Strategy to Foster the Dermal Penetration Efficacy
Source: Pharmaceutics. 2022 May 11;14(5):1039. doi: 10.3390/pharmaceutics14051039 (PMC9144500; doi:10.3390/pharmaceutics14051039)
Supplement: Supplementary file 1 [file pharmaceutics-14-01039-s001.zip › pharmaceutics-1675980-supplementary.pdf]

Particle assisted dermal penetration – a mechanistic proof of concept study

Sabrina Wiemann and Cornelia M. Keck *

Supplementary material – Section S1

All images were subjected to an automated RGB-threshold algorithm. The macros were established with ImageJ software for each part of the study to adjust for the variations in the skin`s autofluorescence. The macros used are provided in table S1.

Table S1: Macros used for the RGB-threshold algorithms.

**Particle assisted dermal penetration with titanium dioxide particles**	**Particle assisted dermal penetration with NLC**	**Particle assisted penetration and addition of humectants**
// Color Thresholder 1.52a	// Color Thresholder 1.52a	// Color Thresholder 1.52a
// Autogenerated macro, single images only!	// Autogenerated macro, single images only!	// Autogenerated macro, single images only!
min=newArray(3);	min=newArray(3);	min=newArray(3);
max=newArray(3);	max=newArray(3);	max=newArray(3);
filter=newArray(3);	filter=newArray(3);	filter=newArray(3);
a=getTitle();	a=getTitle();	a=getTitle();
run("RGB Stack");	run("RGB Stack");	run("RGB Stack");
run("Convert Stack to Images");	run("Convert Stack to Images");	run("Convert Stack to Images");
selectWindow("Red");	selectWindow("Red");	selectWindow("Red");
rename("0");	rename("0");	rename("0");
selectWindow("Green");	selectWindow("Green");	selectWindow("Green");
rename("1");	rename("1");	rename("1");
selectWindow("Blue");	selectWindow("Blue");	selectWindow("Blue");
rename("2");	rename("2");	rename("2");
min[0]=0;	min[0]=0;	min[0]=0;
max[0]=0;	max[0]=0;	max[0]=0;
filter[0]="stop";	filter[0]="stop";	filter[0]="stop";
min[1]=100;	min[1]=60;	min[1]=119;
max[1]=255;	max[1]=255;	max[1]=255;
filter[1]="pass";	filter[1]="pass";	filter[1]="pass";
min[2]=0;	min[2]=0;	min[2]=0;
max[2]=0;	max[2]=0;	max[2]=0;
filter[2]="stop";	filter[2]="stop";	filter[2]="stop";
for (i=0;i<3;i++){	for (i=0;i<3;i++){	for (i=0;i<3;i++){
selectWindow(""+i);	selectWindow(""+i);	selectWindow(""+i);
setThreshold(min[i], max[i]);	setThreshold(min[i], max[i]);	setThreshold(min[i], max[i]);
run("Convert to Mask");	run("Convert to Mask");	run("Convert to Mask");
if (filter[i]=="stop") run("Invert");	if (filter[i]=="stop") run("Invert");	if (filter[i]=="stop") run("Invert");

}<br>imageCalculator("AND create", "0","1");<br>imageCalculator("AND create", "Result of 0","2");<br>for (i=0;i<3;i++){<br>selectWindow(""+i);<br>close();<br>}<br>selectWindow("Result of 0");<br>close();<br>selectWindow("Result of Result of 0");<br>rename(a);<br>// Colour Thresholding-------------<br>run("Invert");	}<br>imageCalculator("AND create", "0","1");<br>imageCalculator("AND create", "Result of 0","2");<br>for (i=0;i<3;i++){<br>selectWindow(""+i);<br>close();<br>}<br>selectWindow("Result of 0");<br>close();<br>selectWindow("Result of Result of 0");<br>rename(a);<br>// Colour Thresholding-------------<br>run("Invert");	}<br>imageCalculator("AND create", "0","1");<br>imageCalculator("AND create", "Result of 0","2");<br>for (i=0;i<3;i++){<br>selectWindow(""+i);<br>close();<br>}<br>selectWindow("Result of 0");<br>close();<br>selectWindow("Result of Result of 0");<br>rename(a);<br>// Colour Thresholding-------------<br>run("Invert");
